# YB-1 drives preneoplastic progression: Insight into opportunities for cancer prevention

**DOI:** 10.18632/oncotarget.276

**Published:** 2011-05-13

**Authors:** Alastair H. Davies, Sandra E. Dunn

**Affiliations:** ^1^ Laboratory of Oncogenomic Research, Departments of Pediatrics and Experimental Medicine, Child and Family Research Institute, University of British Columbia, Vancouver, BC, V5Z 4H4, Canada

**Keywords:** YB-1, HER2, RSK, MAPK signaling, pre-malignancy, Kinex antibody microarray

## Abstract

Surprisingly little is known about the underlying genetic events that trigger the progression of a normal cell into a cancerous cell. We recently developed a YB-1-driven model of pre-malignancy where we uncovered that the oncogene promotes genomic instability through cell cycle checkpoint slippage and centrosome amplification. In this research perspective, we describe a possible mechanism by which YB-1 instigates preneoplastic transformation. Using Kinex antibody microarrays with coverage of 800 proteins, we discovered that pre-malignant cells exhibit deregulated signal transduction along the HER2-MAPK-RSK axis. We will discuss the implications of these finding in regard to early intervention strategies.

## INTRODUCTION

One of the most critical issues facing breast cancer treatment is late detection when the odds of long-term disease free survival are the lowest. The earliest lesions and genetic transitions usually occur years before a tumour is detected by palpation or mammography [1]. While early stage ductal carcinoma *in situ* (DCIS) is considered a non-life-threatening disease with a 10-year survival rate of about 90%, this drops dramatically to well under 10% when the cancer is detected at later stages [2]. This is largely a result of tumour cells disseminating to form micrometastases in distant organs as well as acquiring resistance toward chemotherapeutics [3, 4]. Accordingly, it has become imperative to understand the earliest events that trigger the progression of a normal cell into a malignant cell. The identification of biomarkers and development of selective therapeutics targeting key pathways in pre-malignant cells will represent a holy grail in breast cancer treatment and prevention.

A number of obstacles have hindered the study of early tumour progression. First and foremost is the lack of *in vitro* models. One approach to tackling this problem has been to introduce genes into primary human mammary epithelial cells (HMECs) in an attempt to transform them. Expression of the SV40 large-T antigen, the telomerase catalytic subunit, and H-Ras for example yields HMECs with the capacity to form tumours when injected into immunodeficient mice [5, 6]. Modeling pre-malignancy *in vitro* is complex and has only been made possible in the last decade through the advent of three-dimensional acini cultures. This model provides a context in which it is feasible to identify genes and dissect the mechanisms necessary to produce phenotypic alterations similar to those observed during malignant progression. These can include luminal filling, loss of polarization, and invasive behaviour [7, 8]. Taken together, while progress has been made, work is still required to accurately model pre-malignancy in order to understand the events that drive a cell towards a cancer fate. Moving forward these models can be used to understand the role of oncogenes that are more common to breast cancer.

## YB-1 IN PRE-MALIGNANCY AND BREAST CANCER PREDISPOSITION

The importance of Y-box binding protein-1 (YB-1) in the maintenance of breast cancer cell lines is well documented [9-12]. However, its role in cancer initiation has, until recently, been unappreciated [13]. Our lab developed and characterized an *in vitro* model of pre-malignancy following conditional YB-1 expression in genetically stable HMECs. We discovered that the sole expression of YB-1 was sufficient to prime cells for malignancy by promoting cell cycle checkpoint slippage, which led to numerical and structural chromosomal aberrations. Interestingly, we elucidated that these genetic changes were not stochastic but there was a propensity for *HER2* amplification in a subset of cells [13]. In many respects our model mirrors tumour progression in YB-1 transgenic mice. For instance, the expression of YB-1 both *in vitro* and in mice imposed genetic instability that materialized as centrosome amplification and aneuploidy [13, 14]. The clear advantage to using an *in vitro* model is that it takes only days to promote chromosomal instability whereas generating preneoplastic lesions in YB-1 transgenic mice can take 6-8 months (that is following the time-intensive effort of establishing the transgenic mouse colonies). It also provides a rapid screening platform for identifying agents that may block YB-1-mediated changes at a preneoplastic stage of breast cancer progression.

An immediate question at hand is whether these findings translate into risk associated with the development of breast cancer in women. While it is well established that YB-1 is found in approximately 50% of invasive breast cancers [12], its expression has not been examined to any great depth in DCIS. A large gap in the literature exists with regard to the frequency of YB-1 expression in DCIS and whether it is associated with high-grade lesions and/or the eventual development of invasive carcinomas. In a small study by Dahl and colleagues, YB-1 was expressed in 6/8 DCIS [15]. This line of investigation should be followed up independently and with a larger number of samples. In addition, probing the expression of YB-1 in a breast cancer progression series, such as that developed in the human 21T breast epithelial cells [16] or murine 67NR/4T1 cells [17], will provide much needed insight into the role of the oncogene at each stage of cancer evolution.

Unlike in hereditary breast cancer, the genetic factors that predispose women to spontaneous breast cancer are not well defined. Based on its role in pre-malignancy, we believe that YB-1 could play a fundamental role in predisposing individuals to cancer given the evidence that it instills genomic instability and it has the capacity to transform normal mammary epithelial cells. Typically YB-1 is not expressed in differentiated breast epithelial cells. Yet for reasons that are still not understood, its expression is induced in tumours. It is possible that there is an expansion of progenitor cells that permits the induction of YB-1. This idea arises from the evidence that YB-1 is detectable at low levels in normal mammary progenitor cells in healthy, disease-free women [18]. Perhaps these cells lay dormant for many years until they acquire additional mutations in oncogenes and tumour suppressor genes. Interestingly, p16^INK4a^ has been reported to be repressed in a small subset of cells from women who have undergone reduction mammoplasty [19, 20]. Compelling evidence suggests that these cells are the precursors to malignancy and simply require a second oncogenic hit to become transformed [21]. It will be interesting to ascertain if this population of cells overlaps with the cells expressing YB-1. This is presently an understudied area of research that if substantiated could lead to a new understanding of the etiology of breast cancer and its role in neoplastic progression.

## PRE-MALIGNANT CELLS CAN BE CHARACTERIZED BY DEREGULATED HER2/MAPK/RSK SIGNALING

**Figure 1:**
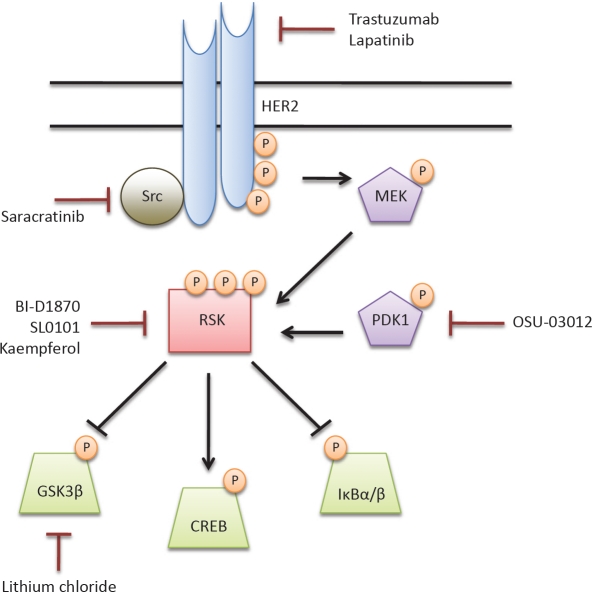
Signal transduction along the MAPK/RSK pathway is altered in pre-malignant cells Using the Kinexus Kinex antibody microarray, we uncovered an enhancement in signal transduction along the MAPK/RSK pathway following YB-1 induction in HMECs. Activation of HER2 led to phosphorylation of MEK and, in turn, RSK. Downstream RSK substrates with a role in cell survival and proliferation demonstrated altered expression and activity. Select therapeutic agents that could inhibit signaling at each level of the MAPK/RSK cascade were identified. The specific phosphorylation sites and the percent change in protein expression following a 72-hour YB-1 induction in HMECs (%CFC) are provided in the table.

To aid in risk assessment and elimination of pre-malignant cells, there has been much interest in uncovering signaling pathways and nodes principal to early transformation. To begin to understand global changes in protein expression following YB-1 expression in HMECs we utilized the Kinexus Kinex antibody microarray platform. This allowed us to query changes in the expression and activity of over 800 proteins concurrently [13]. From this unbiased proteomics array, we have identified an astounding enhancement in signal transduction along the HER2-p90 ribosomal S6 kinase (RSK) arm of the mitogen-activated protein kinase (MAPK) pathway (Figure 1). In support of this, YB-1 seems to focally regulate genes specific to the MAPK family [22]. The MAPK pathway is synonymous with cell proliferation and it is deregulated in nearly one third of human cancers [23]. Despite this, very little is known about the importance of MAPK signaling in pre-malignancy.

*ErbB2* (*HER2*) is an oncogene that leads to the development of mammary tumours in mice [24]. In breast cancer HER2 is overexpressed in about 30% of cases where it is correlated with poor survival [25, 26]. In our model of pre-malignancy, we detected a 196% enhancement in activated HER2^Y1248^, which is associated with cell migration and invasion via activation of PLKγ and the diacylglycerol pathway (Figure 1) [27, 28]. Moreover, we identified a subset of cells with *HER2* amplification; further, stressing the dependence on HER2 signaling in this model [13]. In clinical samples, upregulation of HER2 can be readily detected in breast tissues that demonstrate early signs of transformation but have not been completely transformed [29]. This suggests that overexpression of HER2 is an important initializing event in pre-malignant breast disease. The increased proliferation and cell motility associated with overexpression of the gene are hypothesized to contribute greatly to malignancy. In support of this, patients with low-level *HER2* gene amplification have a two- to three-fold increased risk of developing high-grade inflammatory breast cancer [30]. Importantly, the fact that HER2 activation in pre-malignant clinical samples correlates with our *in vitro* model suggests that we have engineered an accurate representation of pre-malignancy.

The discovery that pre-malignant cells overexpress HER2 provides an opportunity for therapeutic intervention. As both EGFR and HER2 were elevated as a consequence of YB-1 induction in our HMEC model [13], treatment with lapatinib might be a promising strategy for targeting these cells. This second-generation tyrosine kinase inhibitor binds to the ATP-binding pocket of EGFR/HER2 dimers to prevent auto-phosphorylation and, in turn, activation of MAPK signaling [31]. Lapatinib has also been shown to prevent the development of estrogen receptor negative mammary tumourigenesis [32], which is an important step forward given the current lack of therapies for these tumours. Upon further consideration of the signaling events that are altered as a consequence of YB-1 induction, lapatinib may or may not prevent the growth of preneoplastic cells due to changes downstream such as SRC and/or RSK activation.

## SRC ACTIVATION AS AN EARLY EVENT IN NEOPLASTIC CONVERSION

SRC exhibits classic transforming properties by activating signaling through the RAS pathway. The fact that it is activated following YB-1 induction provides further insight into the molecular events that cooperate to achieve cellular transformation and cancer initiation. It has recently been reported that SRC activation is a culprit in mediating resistance to the HER2 targeting agent trastuzumab [33]. SRC is indeed activated by HER2 [34] but also by several other growth factor receptors such as IGF-1R and the Met receptor [35, 36]. Given that it is a convergence point for several RTKs, which may be up-regulated as a consequence of YB-1 induction, it would seem that SRC inhibitors such as saracratinib could potentially be used as a strategy for intervention.

## RSK FUNCTIONS AS A SIGNALING NODE IN PRE-MALIGNANT CELLS

Following YB-1 expression, we measured a 269% increase in activated RSK1/2 phosphorylated at the Ser-380/386 residue (Figure 1). The current dogma of RSK activation suggests that it is first phosphorylated by ERK1/2. This allows for auto-phosphorylation of Ser-380/386, providing a docking site for 3-phosphoinositide-dependent protein kinase 1 (PDK1) and complete RSK activation [37]. The RSKs, which are overexpressed in a plethora of cancers, function as principal signaling nodes orchestrating the expression of transcription factors, anti-apoptotic factors, and cell cycle regulators [38, 39]. Notably, we uncovered significantly altered expression and/or activity of three RSK downstream targets in our pre-malignancy model: cAMP-responsive element binding protein^S129/S133^ (CREB; 71%), glycogen synthase kinase-3β^Y279/Y216^ (GSK3β; 177%), and IκBα/β (-60%). It is probably not a coincidence that these changes function to promote cell survival and drive proliferation. Specifically, repression of GSK3β and the NFκB inhibitor IκBα/β via RSK-mediated phosphorylation would enhance cell cycle progression [40, 41]. On the other hand, CREB phosphorylation would yield increased transcription of pro-survival genes including *Bcl2*, *Bcl-XL*, and *Mcl1*[42].

The identification of RSK1/2 as a central signaling node in pre-malignancy makes it an enticing therapeutic target. To date, a number of ATP competitive inhibitors have been identified against RSKs including BI-D1870, SL0101, and kaempferol; however, none have been tested in clinical trials [38, 39, 43]. Kaempferol, a natural flavonoid, was one of the first RSK inhibitors to be described and has recently been shown to significantly reduce the risk of developing pancreatic cancer [44]. We envision that this could translate into breast cancer where the drug would be used for patients who have been diagnosed with DCIS and are at risk of subsequently developing invasive breast cancer. An alternative approach to targeting RSK activity would be to use small molecule inhibitors against PDK1, as it is an absolute requirement for RSK activation. Our lab has demonstrated that the PDK1 inhibitor OSU-03012 prevented phosphorylation of RSK substrates [45]. Downstream of RSK, one could also indirectly influence signaling by blocking GSK3β with lithium chloride (Figure 1). RSK inhibitors (direct or indirect) could be considered for patients who had tumours with high YB-1 expression in their primary tumour and as such would be at a greater risk of recurrence [12]. These inhibitors could thus be considered for protecting against the development of bilateral breast cancer occurrence.

## THE HER2-RSK-YB-1 AXIS IN TUMOUR-INITIATING CELLS

Recently, considerable research has been focused on dissecting the role of tumour initiating cells (TICs) during cancer progression and relapse. During the development of pre-malignancy in mice, the expression of YB-1 increased the TIC surface markers CD44 and CD49f. Likewise, ectopic expression of YB-1 in HMECs increased CD44 mRNA [10]. It is therefore tempting to speculate that YB-1 plays a prominent role in the emergence of TICs. This is substantiated by the fact that in a ChIP-on-chip analysis YB-1 was found bound to the promoters of stem cell associated genes, notably notch and wnt family numbers [18]. Moreover, our lab made the initial discovery that YB-1 binds to the *HER2* promoter to activate gene transcription [46]. Interestingly, there is a well-established positive correlation between the stem cell marker ALDH and *HER2* overexpression in breast cancer patients [47]. While Wicha and colleagues have characterized HER2 to regulate self-renewal and invasion of human mammary stem cells [48] the mechanism remains elusive. We postulate that as a consequence of HER2 overexpression and MAPK signal transduction, RSK activation and phosphorylation of its downstream targets, including YB-1, promote a TIC phenotype. In support of this hypothesis, the RSK substrate CREB is highly expressed in leukemia stem cells to enhance their proliferation [49, 50]. To deduce whether our pre-malignant model has tumour-initiating capacity, the next logical step will be to inject these cells at limiting dilutions into mice to ascertain if they form tumours. We believe that YB-1 could represent a promising biomarker for the detection of TICs and possibly presents a novel therapeutic targeting opportunity.

## PERSPECTIVE

Given the numerous studies conducted over the past decades it appears that early detection of breast cancer provides the greatest opportunity for a cure. Along with this comes the need to understand what drives these early changes in cellular growth that lead to cancer. Accordingly, there is an insatiable need for *in vitro* models that accurately reproduce malignant progression. While pre-malignant and pre-invasive breast lesions are relatively common only a small percentage progress to high grade invasive breast cancer [51]. Therefore, important biological differences must exist between those that remain stable and those that progress into a cancer. By identifying and treating high-risk pre-malignant lesions there is the potential to prevent the emergence of invasive and metastatic breast cancer. We believe that YB-1 represents one of the most promising biomarkers for identification of pre-malignant cells with strong tumourigenic potential. The addiction of these cells to HER2 and RSK provides a therapeutic strategy for eliminating cancer in its infancy. With the rampant advancement in our understanding of pre-malignancy, the establishment of robust models, and the development of therapeutics that target these cells the potential to prevent breast cancer progression has never been greater.
